# FishSNP: a high quality cross-species SNP database of fishes

**DOI:** 10.1038/s41597-024-03111-8

**Published:** 2024-03-09

**Authors:** Lei Zhang, Heng Li, Mijuan Shi, Keyi Ren, Wanting Zhang, Yingyin Cheng, Yaping Wang, Xiao-Qin Xia

**Affiliations:** 1 State Key Laboratory of Freshwater Ecology and Biotechnology, Hubei Hongshan Laboratory, Key Laboratory of Aquaculture Disease Control, Ministry of Agriculture and Rural Affairs, The Innovation Academy of Seed Design, Institute of Hydrobiology, Chinese Academy of Sciences, Wuhan, 430072 China; 2 College of Advanced Agricultural Sciences, University of Chinese Academy of Sciences, Beijing, 100049 China; 3 College of Fisheries and Life Science, Dalian Ocean University, Dalian, 116023 China

**Keywords:** Data integration, Genetic markers

## Abstract

The progress of aquaculture heavily depends on the efficient utilization of diverse genetic resources to enhance production efficiency and maximize profitability. Single nucleotide polymorphisms (SNPs) have been widely used in the study of aquaculture genomics, genetics, and breeding research since they are the most prevalent molecular markers on the genome. Currently, a large number of SNP markers from cultured fish species are scattered in individual studies, making querying complicated and data reuse problematic. We compiled relevant SNP data from literature and public databases to create a fish SNP database, FishSNP (http://bioinfo.ihb.ac.cn/fishsnp), and also used a unified analysis pipeline to process raw data that the author of the literature did not perform SNP calling on to obtain SNPs with high reliability. This database presently contains 45,690,243 (45 million) nonredundant SNP data for 13 fish species, with 30,288,958 (30 million) of those being high-quality SNPs. The main function of FishSNP is to search, browse, annotate and download SNPs, which provide researchers various and comprehensive associated information.

## Background & Summary

In the post-genomics era, the endeavor to improve the efficiency, sustainability, product quality, and profitability of aquaculture production is increasingly rely on the diverse genetic knowledges obtained from genome study of each aquaculture species^1^. The genome study is frequently based on gene type, gene quantity, and sequence variation. Insertions, deletions, translocations of large and small fragments, polymorphisms of tandem sequence (satellite DNA) repetitions, and single nucleotide polymorphisms (SNPs) are the most common types of sequence variation. SNPs are common in the genome^2^ and easily detectable by high-throughput sequencing, making them the primary molecular markers in the study of fish genome variation^3–5^. The presence of homologous genes or sequences initially complicates allele identification, however the simplicity with which SNPs can be scored promotes allele discrimination^6^. SNP markers have been widely employed in fish disease resistance^7,8^, feed conversion efficiency^9,10^, growth rate^11,12^, muscle yield^13^, reproductive characteristics^14,15^ and tolerance to environmental stressors^16,17^ since the emergence of high-throughput sequencing technology. Similarly, SNP marker-related sequencing data increased rapidly. However, there have been various issues with using SNP markers in fish research.

First, the SNP results of fish research are dispersed throughout the literature, and fish SNP data in public databases are still quite limited. There are now two major databases that contain some fish SNP information: the EMBL-EBI European Variation Archive (EVA, https://www.ebi.ac.uk/eva) and the Animal QTLdb (https://www.animalgenome.org/tools/SNPnmids). EVA is devoted to collecting data on variation types in species other than humans, including SNP information in zebrafish and several non-model fish, with salmon and trout data accounting for more than half of all SNPs^18,19^. EVA primarily collects pertinent SNPs and background information files that authors upload. However, the disclosure of SNP information in studies is often not required, reducing the integrity of EVA data collection. Furthermore, EVA has not confirmed the author’s marker information, and its correctness remains unknown. AnimalQTLdb mostly collects molecular markers from livestock and poultry, however it also has over 60 K rainbow trout SNPs^20,21^. AnimalQTLdb uses EVA as the standard, compares custom markers to EVA, keeps the same SNP site, and inherits with its ID. There are a few SNP databases that focus on one or two specific fish species, for example, SalmonDB for Atlantic salmon and rainbow trout^22^, and SNPfisher for zebrafish^23^. However, for most important economic fish, their SNPs and related information are still scattered in vast mounts of literatures. A fish SNP database that integrates genomic information, annotations, and covers more complete fish species is urgently needed for fish research.

Second, while the issue of SNP marker accuracy is prevalent to some extent, it is an inherent challenge in fish molecular biology research. Most fish genome research is significantly less extensive than that of humans and model animals, and SNP calling is frequently based on rather limited sequencing data. Poor sequencing quality and depth, as well as inappropriate data processing techniques, will inevitably contribute to an increase in false positives in this situation. Some highly similar homologous sequences scattered throughout the genome have only a few single nucleotide differences. These are treated as alleles during SNP calling, resulting in “pseudo-SNP” markers, which is a typical problem when employing SNP markers^24^. Fish commonly experienced two or more rounds whole-genome duplication events, such as common carp, salmon and trout. The duplication event adds to the genome’s complexity and has crucial consequences for evolutionary studies^25–27^, However, it also copies homologous sequences, resulting in additional “pseudo-SNPs”^28^.

In general, synthesis analysis of fish data from multiple research on the same fish species promotes mutual confirmation and bias removal. However, comparing SNP detection results from fish samples is infeasible. The fundamental reason for this is that the most commonly utilized sequencing methods in fish are restriction site-associated DNA sequencing (RAD-seq) that are cost-effective but low genome coverage^29,30^. Most SNP marker sets produced using RAD-seq have inadequate repeatability^31^. In many circumstances, we may identify various sets of SNP loci from separate investigations, different batches of library builds, and even different samples within the same batch, and there is little or even no overlap between these SNP sets. The upgrading of the reference genome and the development of new versions will make SNP marker interaction verification even more challenging.

To address all of the aforementioned issues, this study collected and categorized a large number of fish SNPs and created the Fish SNP database, which provides trustworthy SNP information for fish research. We acquire SNP data from three sources: (1) fish SNP marker data in public databases; (2) SNP data reported in published literature; and (3) SNP markers obtained by processing original literature data using a unified approach. We noticed that some published studies provided raw sequencing data without SNP information during the collection process, so we used the GATK haplotype process with higher accuracy^32–34^ and general hard filtering parameters for SNP marker filtering^35^ to obtain SNP information. We used a unified pipeline to analyze data that the original authors did not call for SNPs, and we retrieved SNPs with excellent reliability. To eliminate “pseudo-SNP” markers, we used the Mendelian segregation ratios test in pedigree data and the Hardy Weinberg equilibrium test in random populations to identify real SNPs. Various published study results were combined to generate a uniform SNP ID that was compatible with numerous genome versions. An annotation tool, utilizing SnpEff^36,37^ to annotate new SNPs, is also accessible to users, facilitating the creation of a complete genome variation profile of fish species. We anticipate that FishSNP will give more comprehensive SNP information for researchers in aquaculture genomics, genetics and breeding.

## Methods

### Data resources of large scale loach

We performed whole genome sequencing on 20 large scale loach. The large-scale loach adults were collected from the Baishazhou Aquatic Product Market in Wuhan, Hubei Province, China. Ten females and three males were selected for breeding on April 30th, 2020. Their offspring were kept at 20 °C, 25 °C, and 30 °C, three different water temperatures in the lab, and fed enough red worms twice a day. The tails of all these samples were preserved in 95% ethanol, and the DNA was extracted using the CTAB protocol. High-throughput pair-end (150 bp/end) whole genome sequencing (DNBSEQ-T7, Illumina Inc.) was performed on ten female parents (BGI, Wuhan, China), and high-throughput pair-end (150 bp/end) whole genome sequencing (BGISEQ-500, Illumina Inc.) was performed on ten offspring reared at 20 °C (BGI, Wuhan, China). All experiments and animal treatments were carried out according to the principles of Animal Care and Use Committee of Institute of Hydrobiology, Chinese Academy of Sciences.

### Other data resources and integration

The study first assessed the value of fish in culture and evolutionary research, and the existing status of fish SNP inclusion in public SNP databases before selecting 13 fish species to collect and organize data. On the one hand, we collected as much SNP-related literature about these fishes as possible and mined SNPs from it as the FishSNP database’s baseline dataset. Simultaneously, we collected VCF files from EVA for the respective species, integrated, and annotated SNP data as an essential component of FishSNP. SNPs from various sources were integrated based on the SNP’s site information or the alignment information of the flanking sequences.

Although some publications include SNP information in the appendix, the formats and reference genomes are often incompatible. The study compared the genome version of the article with the genome version provided in our database for the attachments containing the genome version and SNP site information. If the genome versions are consistent, the SNP attachments were directly converted to VCF files. Otherwise, the SNP’s 150 bp flanking sequences on the original reference genome or the flanking sequences provided in attachments were aligned to the version of genome in FishSNP using bowtie2.0, then the markers’ positions were obtained.

**Fig. 1:**
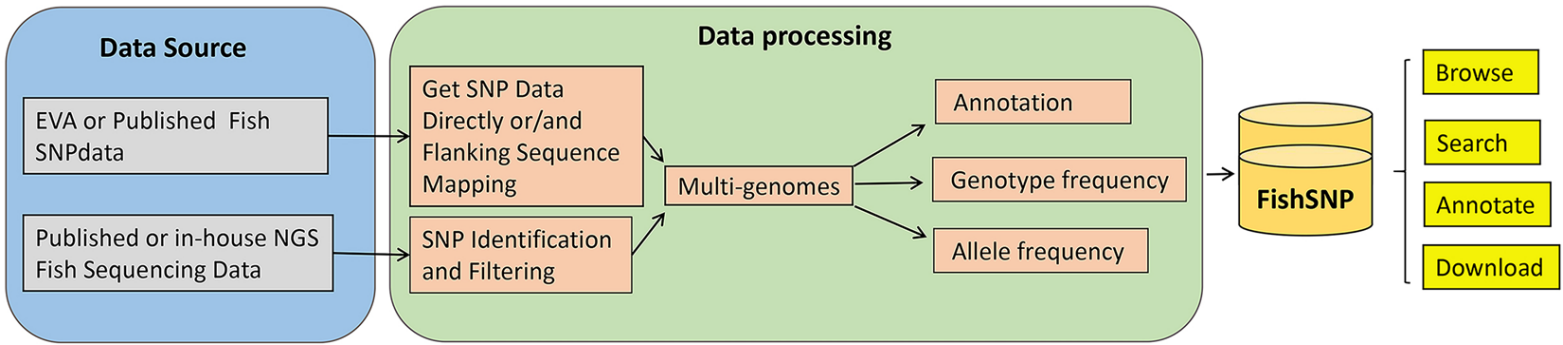
The pipeline for data processing.

SNPs were called using an in-house pipeline after some other literatures provided sequencing data instead of SNPs. We employed a unified SNP calling procedure to retrieve VCF files from the original sequencing data, and we used the corresponding genome and SnpEff software (version 4.3)^36^ for functional annotation (Fig. 1). This procedure necessitated the usage of reference genomes, and we prioritized the genome version that offers structural annotation information on NCBI, the most recently published genome version, and the genome version used by EVA in that order.

Each SNP found in literature was given a unique ID in FishSNP. From left to right, the FishSNP ID consists of three fields. The first two characters “FS” represent the database’s identifier, the next three digits form the serial number of a species ranging from 000 to 999, which is displayed in the “Help” section of the FishSNP website, and the remaining numbers represent the serial number of an SNP. Users can also search all SNPs integrated from the EVA database using EVA’s official ID.

### SNP calling and validation process

Prefetch (2.9.3-1) was used to download the sequencing data, and vdb-validate (2.9.6) was used to evaluate the data integrity, then the sequence data was converted into the original fastq file by fastq-dump (2.9.6). Fastq quality filter (parameter “-q 20 - p 70 -z -Q 33”) was used to clean the original data, and the paired-end data was coupled together using an in-house script. Clean data were aligned to the genome using bowtie2 (2.3.5) with default parameters^38^, followed by a series of procedures from the GATK package (4.1.1.0) (https://github.com/broadinstitute/gatk/releases), including SortSam, Add Or Replace Read Groups, Mark Duplicates, Fix Mate Information, and Haplotype Caller, all of which run with default parameters. Each sample resulted in a gvcf file; Merge the gvcf files of multiple samples (CombineGVCFs, GenotypeGVCFs) and perform hard filtering (Variant Filtration, the parameters are QD < 2.0 || FS > 60.0 || MQ < 35.0 || MQRankSum < −12.5 || ReadPosRankSum < −8.0 || SOR > 3.0)^35,39^. Due to the double-enzyme digestion sequencing principle in data of RAD-seq method, the procedure to mark duplicates (MarkDuplicates) will cause SNPs to be mistakenly deleted by the subsequent process. So MarkDuplicates was applied only to the whole genome re-sequencing data.

We have developed in-house scripts for conducting population tests based on Mendelian segregation ratio and Hardy-Weinberg equilibrium. The script used for the Mendelian segregation ratio test employs a significance cutoff of 0.01 for the P value obtained from the chi-square test. Additionally, we utilized plink1.9^40^ (https://www.cog-genomics.org/plink2) to perform the Hardy-Weinberg equilibrium test, applying the parameter “–hwe 0.000001” to filter error SNPs. Subsequently, quality classification was carried out based on P values. All these scripts have been made publicly available in FishSNP’s download module, or directly at, http://bioinfo.ihb.ac.cn/software/FishSNP and https://github.com/hliihbcas/FishSNP.

## Data Records

All raw data and SNP data of large scale loach were deposited into the GSA database^41–43^ and EVA^44^. All The SNPs collected in this study are summaried by species in Table 1, and the statistics of articles and populations are listed in Supplementary Tables S1 and S2. The datasets have been archived at Figshare^45^. We have organized the data by creating a dedicated folder for each species, and within each species folder, SNP data are stored according to the respective genome version. The “Unmapped” data consists of information gathered from literature sources that cannot be mapped to any currently available genome version. And we also provide symbols to mark the data resources such as EVA. All the literature sources utilized in this research are enumerated in Supplementary Tables S3 and S4. In Supplementary Table S3, we have referenced these papers for accessibility and presented concise descriptions for each of them. In Supplementary Table S4, comprehensive details are presented, including information such as population size, test types, bioproject references, and sequencing methods.

**Table 1:** Summary of SNP numbers of 13 fish species.

Species	Pass (P > 0.01)	Deviation (P ≤ 0.01)	Deviation rate	Untested (including EVA)	Sum
Asian swamp eel	1,824,930	10,760	0.59%	0	1,835,690
Atlantic salmon	3,323,280	730,952	18.03%	7,757,477	11,811,709
barramundi	2,829	0	0.00%	0	2,829
channel catfish	0	0	—	1,242,201	1,242,201
common carp	15,586	2	0.01%	0	15,588
grass carp	202,730	771	0.38%	0	203,501
large scale loach	16,476,754	518,515	3.10%	0	16,995,269
Nile tilapia	6,693,442	1,432,102	17.62%	1,267,701	9,393,245
rainbow trout	57,934	6,220	9.70%	37,780	101,934
rohu labeo	1,528	0	0.00%	2,396,177	2,397,705
silver carp	1,059	82	7.19%	12	1,153
snakehead	3,232	533	14.16%	0	3,765
yellow catfish	1,685,654	0	0.00%	0	1,685,654
Total	30,288,958	2,699,937	8.18%	12,701,348	45,690,243

## Technical Validation

Population tests for Mendelian segregation ratio or Hardy-Weinberg equilibrium rely on the completeness of population information, typically including pedigree details for family-based tests and individual population information for Hardy-Weinberg equilibrium tests. Collecting such information poses significant challenges due to variations in the level of commitment from authors/uploaders and the lack of uniform requirements for data uploading across different journals or public databases. To ensure the clarity regarding the completeness of the collected data, we have presented a comprehensive list of the acquired data (Supplementary Table S4), and provided annotations indicating the availability of different aspects of these datasets in the columns labeled “Description”, “Attachment detail” and “Project detail”.

If a literature provides sequencing data rather than SNPs, the database will only include those datasets that meet the requirements for population tests. So far, the SNP markers directly described in the literatures (or their attachments) do not disclose the genotype of every sample, therefore redoing a population test is unfeasible. It can only be classified as tested or untested based on whether or not the population test was performed as specified in the article. For SNPs with known genotypes of samples, the chi-square test was used on the SNP markers mined by the in-house pipeline to see if they met the Mendelian segregation ratio in pedigree data or the Hardy-Weinberg equilibrium in random group data. Markers with high reliability (P > 0.01) are labeled “Pass” in the database, while “Deviation” (P ≤ 0.01) warrants careful consideration in data analysis, and “Untested” denotes cases where population testing was infeasible due to insufficient essential information (Table 1). If an SNP is found in multiple data sets, the geometric mean of its various P values appears in the SNP’s basic information panel on the database website, whereas the information for a single detection appears in the “Populations” section.

Although EVA does not provide quality control information for all 16,484,194 EVA-derived SNPs, we checked all markers with P values and found 5,264,880 SNPs that were shared with EVA, among which those with better reliability (P > 0.01) occupy 80.35%, a proportion obviously lower than that of the remaining markers (93.98%), suggesting that the quality of SNPs derived from literature and our pipeline is higher than that of EVA.

## Usage Notes

**Fig. 2:**
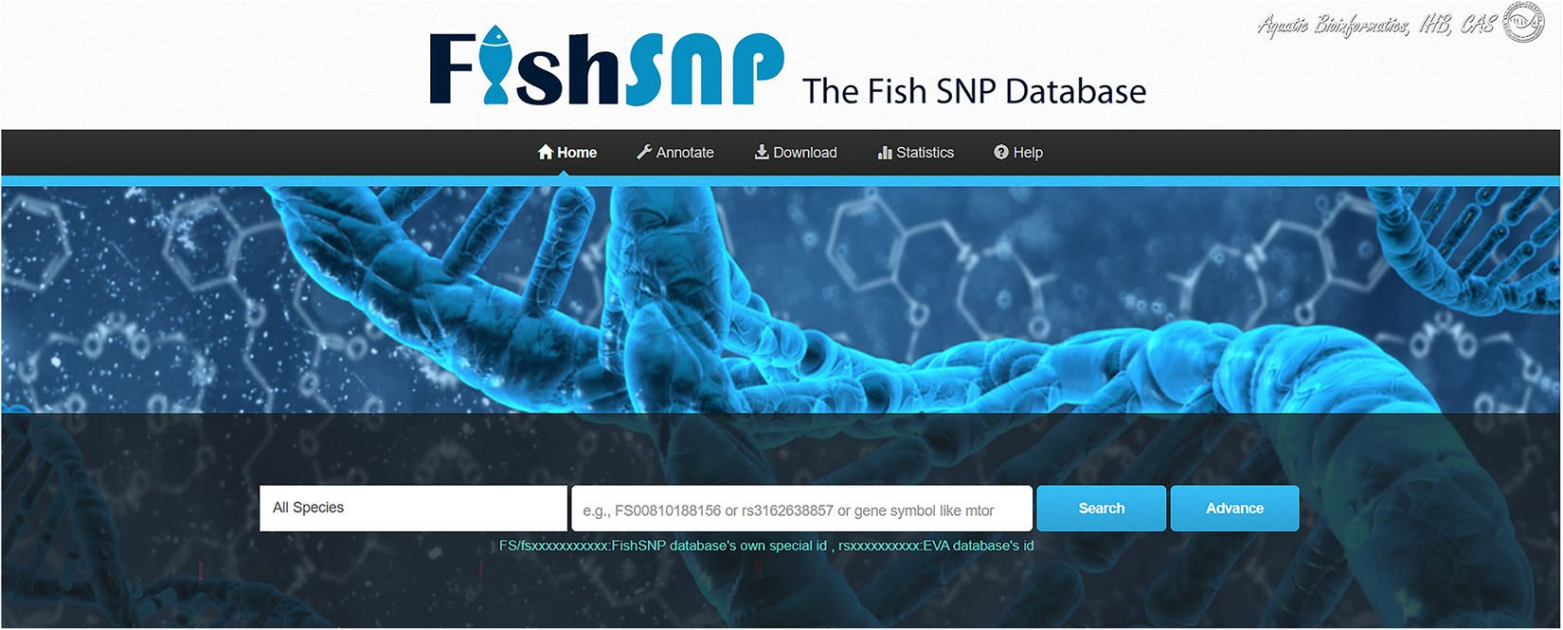
Screenshot of the homepage of the FishSNP database.

We created the FishSNP database to enhance the intuitive and efficient utilization of SNP data. FishSNP encompasses four primary functions for retrieving SNP information: search, browse, annotate, and download (Fig. 2). Users can search and visualize SNP data in various formats, and new SNP data can be subject to be annotated and uploaded to the database. Detailed instructions on how to use these features are available on the “Help” page of the website.

The datasets, which include essential information for each SNP, as well as the P value from the population test, are provided in a VCF format, with detailed explanations of the test results at the beginning of each file. In addition, we have mapped the SNP data to various available genome versions and made them accessible through separate folders under each species, allowing users to select and utilize the data according to their specific needs.

### Supplementary information

Supplementary Information

## Data Availability

The process and script files can be downloaded at http://bioinfo.ihb.ac.cn/software/FishSNP or https://github.com/hliihbcas/FishSNP.
